# Editorial: Insights into Regulatory B Cells

**DOI:** 10.3389/fimmu.2022.903711

**Published:** 2022-04-25

**Authors:** Luman Wang, Damo Xu, Yiwei Chu

**Affiliations:** ^1^ Department of Immunology, School of Basic Medical Sciences, and Institutes of Biomedical Sciences, Fudan University, Shanghai, China; ^2^ State Key Laboratory of Respiratory Disease for Allergy at Shenzhen University, Shenzhen University School of Medicine, Shenzhen, China

**Keywords:** Breg, phenotype, function, applications, organ specific

B cells are typically characterized by their ability to produce antibodies, function as secondary antigen-presenting cells (APCs) and secrete various cytokines. Regulatory B cell (Breg), one of the B cell populations is now widely accepted as an important regulatory subset in the immune system. In this collection, we have received articles from all over the world, including the Dittel lab and the Chu lab who have been working in this field for many years. The published articles and reviews describe current understanding of Breg subsets, functions either in health or diseases, and their therapeutic potential.

Unlike Treg, there are no unique markers or released factors (i.e., specific cytokine profiles) to define Breg. Thus, the term Breg refers to B cells with regulatory functions. In the article of the collection, Fu et al. from Chu lab identified a specific Breg subgroup—CD11b^+^B cells, which specifically secrete IgA in the mucosal immune response and maintain homeostasis. This regulatory function depends on TGF-β signaling pathway. Similarly, the review by Zhong et al. described the regulatory role of IgA^+^B cells in tumor and other diseases. On the contrary, IgA^+^B cells could be induced by tumor microenvironment. Mechanistically, the review by Ding et al. summarized the dysregulation of T follicular helper cells (Tfh)/T follicular regulatory cells (Tfr) in autoimmune diseases are closely associated with Breg under different environments through IL-21, PD-L1/PD-1 and other molecules.

With the aim of discovering the unique phenotypes of Breg, Yang et al. from Lian lab published the article “Organ-Specific Regulatory B Cells Using Single-Cell RNA Sequencing”. They characterized 19 common genes significantly express in Breg cell, including *Fcrl5*, *Zbtb20*, *Ccdc28b*, *Cd9* and *Ptpn22*. Further, they detected the infiltrated Breg in seven tissue or organs and found Breg subgroups existed with different phenotypes. Moreover, they explored *Atf3* molecule as the potential transcription factor to modulate immune response. In addition, Michée-Cospolite et al. demonstrated the role of transcription factors STAT3 and c-MAF in controlling IL-10 production both in murine and human B cells, and they may be the key drivers to initiate the function of Breg. In the review by Neu and Dittel., they provided a complete and in-depth summary of the discovery history of Breg with phenotypic characteristics, functional modes and interaction with Treg.

With the in-depth research in the field of Breg, its applications in diseases have been studied increasingly. Du et al. from Lu lab, reviewed Breg with impaired immunosuppressive function is negatively correlated with Tfh cell in primary Sjögren’s Syndrome (pSS) patients. Further studies have revealed a pivotal role of Breg in constraining Tfh response in autoimmune pathogenesis. This review provides an overview of recent advances in the identification of pathogenic B cell subsets and Breg cells, as well as new development of B-cell targeted therapies in pSS patients. Additionally, Cui et al. reported a retrospective analysis on positron-emission tomography/computed tomography (PET/CT) scans and Breg detection from patients with newly diagnosed multiple myeloma (NDMM) who received bortezomib plus dexamethasone-based (BD) therapy in southwest China. The baseline PET/CT in combination with Bregs can be used as prognosticators. In the article of “Altered Tim-1 and IL-10 Expression in Regulatory B Cell Subsets in Type 1 Diabetes”, Liu et al. showed that altered Tim-1 and IL-10 expression on regulatory B cell in T1D patients. Tim-1 is associated with islet function and blood glucose levels. These findings indicate that Tim-1^+^ and IL-10^+^ Bregs were involved in the pathogenesis of T1D. In drug therapy, Ma et al. found that drugs for treating B cell mediated diseases like Chloroquine (CQ) had a suppressive effect on IL-10 regulatory B cells whereas Granzyme B (GrB) secreting B cells were less affected. Effector functions of B cells such as plasma blast formation and IgG secretion were potently inhibited by CQ. Effector B cells derived from renal transplant patients under immunosuppression circumstance could also be suppressed with diminished IgG secretion by CQ.

In conclusion, this special issue encompasses a collection of articles that highlight the phenotypes, functions, and applications of Breg in regulatory activity by a variety of mechanisms. B cells are no longer considered as effectors in immune response, but are active participants in immune regulation. This new perspective will likely lead to further investigations into regulatory roles for B cells in health and diseases, and to capitalize on the findings to harness B cells functions as therapies. We welcome more immunologists participate in the research of Breg and broaden the field.

## Author Contributions

LW, DX and YC wrote the manuscript. All authors contributed to the article and approved the submitted version.

## Conflict of Interest

The authors declare that the research was conducted in the absence of any commercial or financial relationships that could be construed as a potential conflict of interest.

## Publisher’s Note

All claims expressed in this article are solely those of the authors and do not necessarily represent those of their affiliated organizations, or those of the publisher, the editors and the reviewers. Any product that may be evaluated in this article, or claim that may be made by its manufacturer, is not guaranteed or endorsed by the publisher.

